# μ-2-Amino­terephthalato-κ^2^ *O* ^1^:*O* ^4^-bis­[triphenyl­tin(IV)]

**DOI:** 10.1107/S1600536809043554

**Published:** 2009-10-28

**Authors:** Wenkuan Li, Handong Yin, Lei Dong, Jing Li, Qijun Zhang

**Affiliations:** a College of Chemistry and Chemical Engineering, Liaocheng University, Shandong 252059, People’s Republic of China

## Abstract

The title compound, [Sn_2_(C_6_H_5_)_6_(C_8_H_5_NO_4_)], contains two triphenyl­tin groups bridged by a 2-amino­terephthalate ligand. The two Sn^IV^ centers have similar distorted tetra­hedral coordination geometries. Each Sn^IV^ atom is bonded to three phenyl C atoms and one O atom from a carboxyl­ate group. The other O atom of the carboxyl­ate group has a weak inter­action with the Sn atom. The amino group is disordered over two sites, with site-occupancy factors of 0.779 (11) and 0.221 (11). Intra­molecular N—H⋯O hydrogen bonds are observed.

## Related literature

For general background to organotin compounds, see: Hadjikakou & Hadjiliadis (2009 ▶). For related structures, see: Chandrasekhar *et al.* (2003 ▶); García-Zarracino & Höpfl (2005 ▶); Ma *et al.* (2005 ▶).
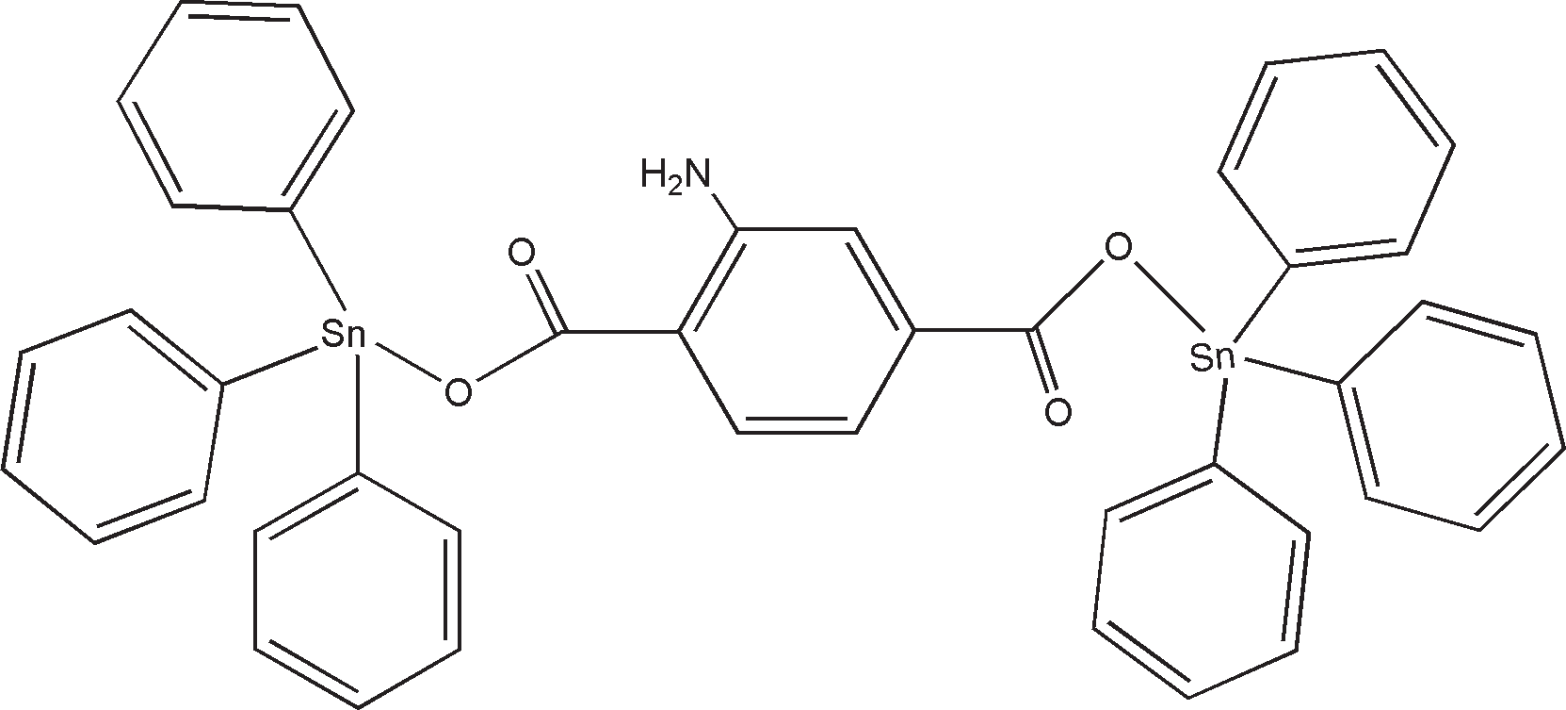


## Experimental

### 

#### Crystal data

[Sn_2_(C_6_H_5_)_6_(C_8_H_5_NO_4_)]
*M* *_r_* = 879.11
Monoclinic, 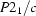

*a* = 16.2933 (14) Å
*b* = 20.6628 (18) Å
*c* = 11.5254 (11) Å
β = 93.032 (1)°
*V* = 3874.8 (6) Å^3^
*Z* = 4
Mo *K*α radiation
μ = 1.33 mm^−1^
*T* = 298 K
0.23 × 0.12 × 0.10 mm

#### Data collection

Siemens SMART 1000 CCD diffractometer
Absorption correction: multi-scan (*SADABS*; Sheldrick, 1996 ▶) *T*_min_ = 0.749, *T*_max_ = 0.878
19986 measured reflections
6817 independent reflections
3977 reflections with *I* > 2σ(*I*)
*R*_int_ = 0.071

#### Refinement

*R*[*F* ^2^ > 2σ(*F* ^2^)] = 0.050
*wR*(*F* ^2^) = 0.118
*S* = 0.99
6817 reflections
470 parameters
H-atom parameters constrained
Δρ_max_ = 0.71 e Å^−3^
Δρ_min_ = −0.51 e Å^−3^

### 

Data collection: *SMART* (Siemens, 1996 ▶); cell refinement: *SAINT* (Siemens, 1996 ▶); data reduction: *SAINT*; program(s) used to solve structure: *SHELXS97* (Sheldrick, 2008 ▶); program(s) used to refine structure: *SHELXL97* (Sheldrick, 2008 ▶); molecular graphics: *SHELXTL* (Sheldrick, 2008 ▶); software used to prepare material for publication: *SHELXTL*.

## Supplementary Material

Crystal structure: contains datablocks I, global. DOI: 10.1107/S1600536809043554/hy2231sup1.cif

Structure factors: contains datablocks I. DOI: 10.1107/S1600536809043554/hy2231Isup2.hkl

Additional supplementary materials:  crystallographic information; 3D view; checkCIF report

## Figures and Tables

**Table 1:** Selected bond lengths (Å)

Sn1—O1	2.069 (4)
Sn1—O2	2.810 (5)
Sn1—C9	2.137 (6)
Sn1—C15	2.147 (6)
Sn1—C21	2.134 (6)
Sn2—O3	2.074 (4)
Sn2—O4	2.794 (5)
Sn2—C27	2.122 (6)
Sn2—C33	2.135 (6)
Sn2—C39	2.140 (6)

**Table 2:** Hydrogen-bond geometry (Å, °)

*D*—H⋯*A*	*D*—H	H⋯*A*	*D*⋯*A*	*D*—H⋯*A*
N1—H1*B*⋯O2	0.86	2.02	2.683 (9)	133
N1′—H1′2⋯O4	0.86	1.99	2.63 (3)	130

**Fig. 1.**
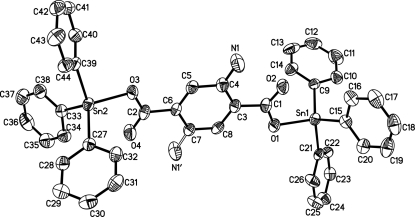
Molecular structure of the title compound, showing 50% probability displacement ellipsoids. H atoms have been omitted for clarity. The amino group is disordered over two sites, N1 and N1'.

## supplementary crystallographic information

### Comment

Tin complexes have attracted much attention for their antitumor properties. Recently, it is noticeable that organotin compounds occupy an important place in cancer chemotherapy (Hadjikakou & Hadjiliadis, 2009). In the last few years, a large number of organotin compounds have been prepared and structurally characterized (Chandrasekhar *et al.*, 2003; García-Zarracino & Höpfl, 2005; Ma *et al.*, 2005). As part of the ongoing study, we report here the synthesis and crystal structure of the title compound.

The title compound possesses a monomeric structure in which two triphenyltin groups are linked together by a 2-aminoterephthalate ligand (Fig. 1). The Sn1—O1 and Sn2—O3 bond distances [2.069 (4) and 2.074 (4) Å] lie in the range of 2.038 (9)–2.115 (6) Å that has been reported as the Sn—O covalent bond length, which proves that the O atoms are coordinated to the Sn atoms by a strong chemical bond. The distances Sn1—O2 [2.810 (5) Å] and Sn2—O4 [2.794 (5) Å] indicate weak interactions between the carbonyl O atoms and the Sn atoms (Table 1). The two Sn centers have similar coordination geometries, each Sn center displaying a tetrahedral coordination environment. The amino group on the benzene-ring is disordered over two positions. Intramolecular hydrogen bonds, N1—H1B···O2 and N1'—H1'2···O4 are observed in the crystal structrue (Table 2).

### Experimental

The reaction was carried out under a nitrogen atmosphere. 2-Aminoterephthalic acid (1 mmol) and sodium ethoxide (2.2 mmol) were added to a stirring solution of benzene/methanol (30 ml) in a Schlenk flask and stirred for 0.5 h. Triphenyltin chloride (2 mmol) was then added to the reactor and the reaction mixture was stirred for 6 h at 323 K. The resulting clear solution was evaporated under vacuum. The product was crystallized from a solution of dichloromethane/methanol (*v*/*v* 1:1) to yield yellow blocks of the title compound (yield 85%; m.p. 453 K). Analysis, calculated for C_44_H_35_NO_4_Sn_2_: C 60.11, H 4.01, N 1.59, O 7.28, Sn 27.00%; found: C 60.09, H 4.00, N 1.60, O 7.30, Sn 27.01%.

### Refinement

The atom N1 was found to be disordered over two sites, and the ratio of the occupancy factors was refined to 0.779 (11):0.221 (11) for N1 and N1'. All H atoms were positioned geometrically and refined as riding, with C—H = 0.93, and N—H = 0.86 Å, and with *U*_iso_(H) = 1.2*U*_eq_(C, N).

### Crystal data

**Table:**

[Sn_2_(C_6_H_5_)_6_(C_8_H_5_NO_4_)]	*F*(000) = 1752
*M**_r_* = 879.11	*D*_x_ = 1.507 Mg m^−^^3^
Monoclinic, *P*2_1_/*c*	Mo *K*α radiation, λ = 0.71073 Å
Hall symbol: -P 2ybc	Cell parameters from 4322 reflections
*a* = 16.2933 (14) Å	θ = 2.5–25.1°
*b* = 20.6628 (18) Å	µ = 1.33 mm^−^^1^
*c* = 11.5254 (11) Å	*T* = 298 K
β = 93.032 (1)°	Needle, yellow
*V* = 3874.8 (6) Å^3^	0.23 × 0.12 × 0.10 mm
*Z* = 4	

### Data collection

**Table:**

Siemens SMART 1000 CCD diffractometer	6817 independent reflections
Radiation source: fine-focus sealed tube	3977 reflections with *I* > 2σ(*I*)
graphite	*R*_int_ = 0.071
φ and ω scans	θ_max_ = 25.0°, θ_min_ = 1.6°
Absorption correction: multi-scan (*SADABS*; Sheldrick, 1996)	*h* = −19→18
*T*_min_ = 0.749, *T*_max_ = 0.878	*k* = −13→24
19986 measured reflections	*l* = −13→13

### Refinement

**Table:**

Refinement on *F*^2^	Primary atom site location: structure-invariant direct methods
Least-squares matrix: full	Secondary atom site location: difference Fourier map
*R*[*F*^2^ > 2σ(*F*^2^)] = 0.050	Hydrogen site location: inferred from neighbouring sites
*wR*(*F*^2^) = 0.118	H-atom parameters constrained
*S* = 0.99	*w* = 1/[σ^2^(*F*_o_^2^) + (0.049*P*)^2^] where *P* = (*F*_o_^2^ + 2*F*_c_^2^)/3
6817 reflections	(Δ/σ)_max_ = 0.001
470 parameters	Δρ_max_ = 0.71 e Å^−^^3^
0 restraints	Δρ_min_ = −0.51 e Å^−^^3^

### Fractional atomic coordinates and isotropic or equivalent isotropic displacement parameters (Å^2^)

**Table:**

	*x*	*y*	*z*	*U*_iso_*/*U*_eq_	Occ. (<1)
Sn1	0.10099 (2)	0.12890 (2)	0.47025 (4)	0.05219 (15)	
Sn2	0.38411 (2)	0.62318 (2)	0.50071 (4)	0.05156 (15)	
N1	0.2956 (6)	0.3125 (4)	0.2880 (8)	0.114 (4)	0.779 (11)
H1A	0.3287	0.3315	0.2434	0.137*	0.779 (11)
H1B	0.2821	0.2727	0.2755	0.137*	0.779 (11)
N1'	0.1716 (19)	0.4464 (14)	0.651 (3)	0.115 (15)	0.221 (11)
H1'1	0.1391	0.4264	0.6957	0.137*	0.221 (11)
H1'2	0.1840	0.4863	0.6649	0.137*	0.221 (11)
O1	0.1354 (3)	0.2228 (2)	0.5117 (4)	0.0692 (13)	
O2	0.2105 (3)	0.2097 (2)	0.3578 (4)	0.0795 (15)	
O3	0.3397 (3)	0.5353 (2)	0.4374 (4)	0.0675 (13)	
O4	0.2656 (3)	0.5431 (2)	0.5924 (5)	0.0835 (16)	
C1	0.1864 (4)	0.2446 (3)	0.4355 (7)	0.0638 (19)	
C2	0.2869 (4)	0.5114 (3)	0.5085 (7)	0.0607 (18)	
C3	0.2115 (4)	0.3142 (3)	0.4498 (6)	0.0562 (17)	
C4	0.2659 (4)	0.3438 (3)	0.3745 (6)	0.0632 (19)	
H4	0.2866	0.3209	0.3131	0.076*	0.221 (11)
C5	0.2882 (4)	0.4090 (3)	0.3949 (6)	0.0586 (17)	
H5	0.3240	0.4290	0.3460	0.070*	
C6	0.2583 (4)	0.4436 (3)	0.4849 (6)	0.0516 (16)	
C7	0.2036 (4)	0.4148 (3)	0.5592 (6)	0.0596 (18)	
H7	0.1825	0.4385	0.6195	0.072*	0.779 (11)
C8	0.1815 (4)	0.3513 (3)	0.5423 (6)	0.0582 (17)	
H8	0.1459	0.3321	0.5926	0.070*	
C9	0.0258 (4)	0.1346 (3)	0.3130 (5)	0.0515 (16)	
C10	−0.0170 (5)	0.0805 (4)	0.2715 (6)	0.077 (2)	
H10	−0.0089	0.0410	0.3093	0.093*	
C11	−0.0726 (5)	0.0839 (5)	0.1734 (8)	0.104 (3)	
H11	−0.1012	0.0473	0.1472	0.124*	
C12	−0.0836 (6)	0.1415 (6)	0.1178 (7)	0.102 (3)	
H12	−0.1205	0.1445	0.0537	0.122*	
C13	−0.0408 (6)	0.1952 (5)	0.1552 (7)	0.093 (3)	
H13	−0.0473	0.2340	0.1151	0.112*	
C14	0.0124 (4)	0.1916 (4)	0.2533 (6)	0.070 (2)	
H14	0.0397	0.2288	0.2793	0.084*	
C15	0.2028 (3)	0.0628 (3)	0.4790 (6)	0.0526 (16)	
C16	0.2554 (4)	0.0572 (4)	0.3900 (6)	0.073 (2)	
H16	0.2516	0.0863	0.3283	0.087*	
C17	0.3143 (5)	0.0082 (5)	0.3920 (8)	0.090 (3)	
H17	0.3491	0.0039	0.3311	0.108*	
C18	0.3205 (5)	−0.0344 (4)	0.4860 (10)	0.094 (3)	
H18	0.3595	−0.0673	0.4880	0.113*	
C19	0.2698 (5)	−0.0278 (4)	0.5741 (9)	0.095 (3)	
H19	0.2750	−0.0561	0.6367	0.114*	
C20	0.2104 (4)	0.0199 (3)	0.5737 (7)	0.069 (2)	
H20	0.1759	0.0236	0.6352	0.082*	
C21	0.0269 (4)	0.1116 (3)	0.6144 (5)	0.0509 (15)	
C22	−0.0530 (4)	0.0879 (3)	0.6002 (6)	0.0553 (16)	
H22	−0.0750	0.0800	0.5255	0.066*	
C23	−0.1008 (4)	0.0757 (3)	0.6940 (6)	0.0638 (19)	
H23	−0.1540	0.0600	0.6812	0.077*	
C24	−0.0702 (4)	0.0868 (4)	0.8053 (6)	0.072 (2)	
H24	−0.1019	0.0786	0.8684	0.087*	
C25	0.0088 (5)	0.1104 (4)	0.8218 (6)	0.084 (2)	
H25	0.0301	0.1182	0.8970	0.101*	
C26	0.0568 (4)	0.1227 (4)	0.7297 (6)	0.072 (2)	
H26	0.1098	0.1386	0.7437	0.086*	
C27	0.4553 (3)	0.6039 (3)	0.6564 (5)	0.0492 (15)	
C28	0.4742 (4)	0.6534 (4)	0.7344 (6)	0.0687 (19)	
H28	0.4552	0.6949	0.7164	0.082*	
C29	0.5197 (5)	0.6444 (4)	0.8370 (7)	0.087 (2)	
H29	0.5317	0.6792	0.8863	0.104*	
C30	0.5473 (5)	0.5828 (5)	0.8659 (7)	0.083 (2)	
H30	0.5773	0.5756	0.9356	0.099*	
C31	0.5300 (4)	0.5320 (4)	0.7901 (7)	0.079 (2)	
H31	0.5488	0.4906	0.8090	0.094*	
C32	0.4848 (4)	0.5421 (3)	0.6863 (6)	0.0643 (19)	
H32	0.4741	0.5075	0.6360	0.077*	
C33	0.2954 (4)	0.6970 (3)	0.5277 (5)	0.0516 (16)	
C34	0.2495 (4)	0.6946 (3)	0.6257 (6)	0.0648 (19)	
H34	0.2551	0.6595	0.6760	0.078*	
C35	0.1955 (5)	0.7441 (4)	0.6493 (7)	0.082 (2)	
H35	0.1642	0.7418	0.7144	0.099*	
C36	0.1885 (5)	0.7961 (4)	0.5766 (9)	0.093 (3)	
H36	0.1524	0.8293	0.5926	0.112*	
C37	0.2348 (5)	0.8000 (4)	0.4785 (8)	0.091 (3)	
H37	0.2301	0.8358	0.4296	0.109*	
C38	0.2879 (4)	0.7501 (3)	0.4550 (6)	0.068 (2)	
H38	0.3188	0.7522	0.3895	0.082*	
C39	0.4583 (4)	0.6455 (3)	0.3583 (5)	0.0500 (16)	
C40	0.4259 (4)	0.6553 (3)	0.2455 (6)	0.0621 (18)	
H40	0.3693	0.6535	0.2304	0.075*	
C41	0.4768 (5)	0.6679 (4)	0.1550 (6)	0.075 (2)	
H41	0.4540	0.6732	0.0799	0.090*	
C42	0.5597 (5)	0.6725 (4)	0.1753 (7)	0.083 (2)	
H42	0.5934	0.6811	0.1145	0.100*	
C43	0.5933 (5)	0.6645 (4)	0.2864 (7)	0.085 (2)	
H43	0.6497	0.6688	0.3008	0.102*	
C44	0.5436 (4)	0.6500 (4)	0.3774 (6)	0.068 (2)	
H44	0.5673	0.6433	0.4517	0.082*	

### Atomic displacement parameters (Å^2^)

**Table:**

	*U*^11^	*U*^22^	*U*^33^	*U*^12^	*U*^13^	*U*^23^
Sn1	0.0469 (3)	0.0480 (3)	0.0620 (3)	−0.0044 (2)	0.0065 (2)	0.0041 (2)
Sn2	0.0482 (3)	0.0452 (3)	0.0621 (3)	−0.0012 (2)	0.0105 (2)	0.0023 (2)
N1	0.135 (8)	0.076 (6)	0.142 (9)	−0.035 (6)	0.090 (7)	−0.034 (6)
N1'	0.13 (3)	0.08 (2)	0.14 (3)	−0.04 (2)	0.09 (2)	−0.03 (2)
O1	0.077 (3)	0.055 (3)	0.077 (3)	−0.020 (3)	0.015 (3)	0.003 (2)
O2	0.089 (4)	0.056 (3)	0.096 (4)	−0.015 (3)	0.028 (3)	−0.006 (3)
O3	0.077 (3)	0.052 (3)	0.075 (3)	−0.023 (3)	0.011 (3)	−0.005 (2)
O4	0.089 (4)	0.062 (3)	0.103 (4)	−0.017 (3)	0.033 (3)	−0.015 (3)
C1	0.059 (4)	0.050 (4)	0.083 (5)	−0.006 (4)	0.007 (4)	0.004 (4)
C2	0.050 (4)	0.048 (4)	0.084 (5)	−0.011 (4)	0.005 (4)	0.009 (4)
C3	0.051 (4)	0.049 (4)	0.069 (5)	−0.005 (3)	0.000 (3)	0.005 (3)
C4	0.055 (4)	0.057 (4)	0.080 (5)	−0.009 (4)	0.019 (4)	−0.001 (4)
C5	0.052 (4)	0.057 (4)	0.067 (5)	−0.015 (4)	0.013 (3)	0.007 (4)
C6	0.051 (4)	0.041 (4)	0.063 (4)	−0.004 (3)	0.005 (3)	0.008 (3)
C7	0.062 (4)	0.054 (4)	0.063 (4)	−0.018 (4)	0.013 (3)	0.004 (4)
C8	0.052 (4)	0.060 (4)	0.064 (4)	−0.011 (4)	0.013 (3)	0.018 (4)
C9	0.051 (4)	0.054 (4)	0.050 (4)	0.009 (3)	0.008 (3)	0.006 (3)
C10	0.091 (5)	0.065 (5)	0.073 (5)	0.020 (5)	−0.013 (4)	−0.009 (4)
C11	0.104 (7)	0.108 (8)	0.095 (7)	0.012 (6)	−0.026 (6)	−0.026 (6)
C12	0.103 (7)	0.141 (9)	0.060 (5)	0.042 (7)	−0.009 (5)	0.001 (6)
C13	0.095 (7)	0.121 (8)	0.065 (6)	0.041 (6)	0.016 (5)	0.038 (5)
C14	0.064 (4)	0.071 (5)	0.077 (5)	0.011 (4)	0.013 (4)	0.016 (4)
C15	0.034 (3)	0.059 (4)	0.065 (4)	−0.007 (3)	0.001 (3)	−0.007 (4)
C16	0.056 (4)	0.078 (5)	0.083 (5)	−0.001 (4)	0.003 (4)	−0.017 (4)
C17	0.058 (5)	0.094 (7)	0.119 (8)	0.000 (5)	0.017 (5)	−0.031 (6)
C18	0.060 (5)	0.071 (6)	0.149 (9)	0.016 (5)	−0.012 (6)	−0.025 (6)
C19	0.070 (5)	0.067 (6)	0.144 (9)	0.004 (5)	−0.018 (6)	0.012 (6)
C20	0.056 (4)	0.065 (5)	0.085 (5)	−0.004 (4)	−0.001 (4)	0.003 (4)
C21	0.050 (3)	0.040 (4)	0.063 (4)	−0.010 (3)	−0.001 (3)	0.010 (3)
C22	0.053 (4)	0.057 (4)	0.054 (4)	−0.009 (4)	−0.004 (3)	0.003 (3)
C23	0.044 (4)	0.064 (5)	0.084 (5)	−0.006 (4)	0.007 (4)	−0.002 (4)
C24	0.069 (5)	0.083 (5)	0.066 (5)	−0.008 (5)	0.020 (4)	−0.013 (4)
C25	0.081 (5)	0.110 (7)	0.062 (5)	−0.021 (5)	0.010 (4)	−0.027 (5)
C26	0.055 (4)	0.094 (6)	0.066 (4)	−0.021 (4)	0.005 (3)	−0.025 (5)
C27	0.041 (3)	0.053 (4)	0.055 (4)	−0.001 (3)	0.015 (3)	0.002 (3)
C28	0.085 (5)	0.057 (4)	0.065 (5)	0.005 (4)	0.008 (4)	0.001 (4)
C29	0.100 (6)	0.090 (6)	0.069 (5)	−0.003 (5)	0.000 (5)	−0.005 (5)
C30	0.079 (5)	0.103 (7)	0.066 (5)	0.004 (6)	−0.002 (4)	0.016 (5)
C31	0.073 (5)	0.067 (5)	0.095 (6)	0.015 (5)	0.003 (5)	0.025 (5)
C32	0.064 (4)	0.051 (4)	0.078 (5)	0.010 (4)	0.003 (4)	0.003 (4)
C33	0.044 (3)	0.051 (4)	0.060 (4)	0.003 (3)	−0.002 (3)	−0.001 (3)
C34	0.066 (4)	0.068 (5)	0.061 (4)	0.010 (4)	0.009 (4)	−0.003 (4)
C35	0.076 (5)	0.085 (6)	0.086 (6)	0.021 (5)	0.015 (4)	−0.006 (5)
C36	0.078 (6)	0.075 (6)	0.125 (8)	0.033 (5)	−0.002 (6)	−0.030 (6)
C37	0.108 (7)	0.061 (5)	0.102 (7)	0.019 (5)	−0.002 (6)	0.011 (5)
C38	0.070 (5)	0.058 (4)	0.076 (5)	0.009 (4)	0.004 (4)	−0.004 (4)
C39	0.050 (4)	0.038 (3)	0.064 (4)	0.001 (3)	0.018 (3)	0.005 (3)
C40	0.059 (4)	0.064 (4)	0.064 (5)	0.001 (4)	0.004 (4)	−0.004 (4)
C41	0.086 (6)	0.085 (6)	0.055 (5)	−0.013 (5)	0.005 (4)	0.004 (4)
C42	0.090 (6)	0.079 (6)	0.083 (6)	−0.008 (5)	0.034 (5)	0.005 (5)
C43	0.059 (5)	0.099 (7)	0.099 (7)	−0.007 (5)	0.016 (4)	0.017 (5)
C44	0.057 (4)	0.079 (5)	0.070 (5)	−0.009 (4)	0.009 (4)	0.012 (4)

### Geometric parameters (Å, °)

**Table:**

Sn1—O1	2.069 (4)	C18—C19	1.349 (11)
Sn1—O2	2.810 (5)	C18—H18	0.9300
Sn1—C9	2.137 (6)	C19—C20	1.381 (10)
Sn1—C15	2.147 (6)	C19—H19	0.9300
Sn1—C21	2.134 (6)	C20—H20	0.9300
Sn2—O3	2.074 (4)	C21—C22	1.392 (8)
Sn2—O4	2.794 (5)	C21—C26	1.410 (8)
Sn2—C27	2.122 (6)	C22—C23	1.389 (8)
Sn2—C33	2.135 (6)	C22—H22	0.9300
Sn2—C39	2.140 (6)	C23—C24	1.371 (9)
N1—C4	1.303 (9)	C23—H23	0.9300
N1—H1A	0.8600	C24—C25	1.380 (9)
N1—H1B	0.8600	C24—H24	0.9300
N1'—C7	1.37 (3)	C25—C26	1.375 (9)
N1'—H1'1	0.8600	C25—H25	0.9300
N1'—H1'2	0.8600	C26—H26	0.9300
O1—C1	1.320 (8)	C27—C28	1.387 (9)
O2—C1	1.230 (8)	C27—C32	1.401 (8)
O3—C2	1.315 (8)	C28—C29	1.374 (10)
O4—C2	1.234 (8)	C28—H28	0.9300
C1—C3	1.502 (9)	C29—C30	1.385 (11)
C2—C6	1.495 (9)	C29—H29	0.9300
C3—C4	1.412 (8)	C30—C31	1.386 (10)
C3—C8	1.420 (9)	C30—H30	0.9300
C4—C5	1.412 (9)	C31—C32	1.387 (9)
C4—H4	0.9300	C31—H31	0.9300
C5—C6	1.371 (8)	C32—H32	0.9300
C5—H5	0.9300	C33—C38	1.382 (9)
C6—C7	1.401 (8)	C33—C34	1.388 (8)
C7—C8	1.372 (9)	C34—C35	1.385 (9)
C7—H7	0.9300	C34—H34	0.9300
C8—H8	0.9300	C35—C36	1.364 (11)
C9—C14	1.375 (9)	C35—H35	0.9300
C9—C10	1.390 (9)	C36—C37	1.394 (11)
C10—C11	1.413 (10)	C36—H36	0.9300
C10—H10	0.9300	C37—C38	1.382 (10)
C11—C12	1.360 (12)	C37—H37	0.9300
C11—H11	0.9300	C38—H38	0.9300
C12—C13	1.367 (12)	C39—C40	1.392 (8)
C12—H12	0.9300	C39—C44	1.398 (8)
C13—C14	1.389 (10)	C40—C41	1.390 (9)
C13—H13	0.9300	C40—H40	0.9300
C14—H14	0.9300	C41—C42	1.363 (10)
C15—C16	1.376 (9)	C41—H41	0.9300
C15—C20	1.406 (9)	C42—C43	1.375 (10)
C16—C17	1.395 (10)	C42—H42	0.9300
C16—H16	0.9300	C43—C44	1.391 (9)
C17—C18	1.396 (12)	C43—H43	0.9300
C17—H17	0.9300	C44—H44	0.9300
			
O1—Sn1—C21	97.7 (2)	C16—C17—C18	119.5 (8)
O1—Sn1—C9	106.3 (2)	C16—C17—H17	120.3
C21—Sn1—C9	110.3 (2)	C18—C17—H17	120.3
O1—Sn1—C15	112.7 (2)	C19—C18—C17	119.8 (9)
C21—Sn1—C15	108.9 (2)	C19—C18—H18	120.1
C9—Sn1—C15	118.8 (2)	C17—C18—H18	120.1
O1—Sn1—O2	51.56 (16)	C18—C19—C20	121.8 (9)
C21—Sn1—O2	149.03 (19)	C18—C19—H19	119.1
C9—Sn1—O2	85.7 (2)	C20—C19—H19	119.1
C15—Sn1—O2	83.8 (2)	C19—C20—C15	119.0 (8)
O3—Sn2—C27	107.3 (2)	C19—C20—H20	120.5
O3—Sn2—C33	116.7 (2)	C15—C20—H20	120.5
C27—Sn2—C33	110.8 (2)	C22—C21—C26	116.2 (6)
O3—Sn2—C39	96.9 (2)	C22—C21—Sn1	122.1 (5)
C27—Sn2—C39	112.5 (2)	C26—C21—Sn1	121.7 (4)
C33—Sn2—C39	111.9 (2)	C23—C22—C21	122.2 (6)
O3—Sn2—O4	51.58 (16)	C23—C22—H22	118.9
C27—Sn2—O4	85.79 (19)	C21—C22—H22	118.9
C33—Sn2—O4	83.2 (2)	C24—C23—C22	120.4 (6)
C39—Sn2—O4	148.0 (2)	C24—C23—H23	119.8
C4—N1—H1A	120.0	C22—C23—H23	119.8
C4—N1—H1B	120.0	C23—C24—C25	118.6 (7)
H1A—N1—H1B	120.0	C23—C24—H24	120.7
C7—N1'—H1'1	120.0	C25—C24—H24	120.7
C7—N1'—H1'2	120.0	C26—C25—C24	121.5 (7)
H1'1—N1'—H1'2	120.0	C26—C25—H25	119.2
C1—O1—Sn1	109.8 (4)	C24—C25—H25	119.2
C1—O2—Sn1	77.2 (4)	C25—C26—C21	121.1 (6)
C2—O3—Sn2	109.7 (4)	C25—C26—H26	119.5
C2—O4—Sn2	77.8 (4)	C21—C26—H26	119.5
O2—C1—O1	121.4 (6)	C28—C27—C32	116.9 (6)
O2—C1—C3	123.1 (6)	C28—C27—Sn2	120.1 (5)
O1—C1—C3	115.5 (7)	C32—C27—Sn2	123.0 (5)
O4—C2—O3	120.6 (6)	C29—C28—C27	123.3 (7)
O4—C2—C6	122.8 (6)	C29—C28—H28	118.4
O3—C2—C6	116.5 (7)	C27—C28—H28	118.4
C4—C3—C8	118.7 (6)	C28—C29—C30	119.0 (8)
C4—C3—C1	121.5 (6)	C28—C29—H29	120.5
C8—C3—C1	119.8 (6)	C30—C29—H29	120.5
N1—C4—C5	119.9 (7)	C29—C30—C31	119.5 (7)
N1—C4—C3	121.7 (7)	C29—C30—H30	120.3
C5—C4—C3	118.4 (7)	C31—C30—H30	120.3
N1—C4—H4	0.9	C30—C31—C32	120.7 (7)
C5—C4—H4	120.8	C30—C31—H31	119.6
C3—C4—H4	120.8	C32—C31—H31	119.6
C6—C5—C4	121.7 (6)	C31—C32—C27	120.6 (7)
C6—C5—H5	119.2	C31—C32—H32	119.7
C4—C5—H5	119.2	C27—C32—H32	119.7
C5—C6—C7	120.3 (6)	C38—C33—C34	119.1 (6)
C5—C6—C2	120.4 (6)	C38—C33—Sn2	121.2 (5)
C7—C6—C2	119.3 (6)	C34—C33—Sn2	119.4 (5)
C8—C7—N1'	117.2 (13)	C35—C34—C33	120.6 (7)
C8—C7—C6	119.4 (7)	C35—C34—H34	119.7
N1'—C7—C6	123.4 (13)	C33—C34—H34	119.7
C8—C7—H7	120.3	C36—C35—C34	119.6 (8)
N1'—C7—H7	3.3	C36—C35—H35	120.2
C6—C7—H7	120.3	C34—C35—H35	120.2
C7—C8—C3	121.6 (6)	C35—C36—C37	120.9 (8)
C7—C8—H8	119.2	C35—C36—H36	119.6
C3—C8—H8	119.2	C37—C36—H36	119.6
C14—C9—C10	116.9 (6)	C38—C37—C36	119.2 (8)
C14—C9—Sn1	122.7 (5)	C38—C37—H37	120.4
C10—C9—Sn1	120.1 (5)	C36—C37—H37	120.4
C9—C10—C11	121.6 (7)	C37—C38—C33	120.6 (7)
C9—C10—H10	119.2	C37—C38—H38	119.7
C11—C10—H10	119.2	C33—C38—H38	119.7
C12—C11—C10	118.9 (9)	C40—C39—C44	117.5 (6)
C12—C11—H11	120.6	C40—C39—Sn2	123.1 (5)
C10—C11—H11	120.6	C44—C39—Sn2	119.4 (5)
C11—C12—C13	120.7 (8)	C41—C40—C39	121.1 (6)
C11—C12—H12	119.7	C41—C40—H40	119.5
C13—C12—H12	119.7	C39—C40—H40	119.5
C12—C13—C14	119.9 (8)	C42—C41—C40	120.6 (7)
C12—C13—H13	120.0	C42—C41—H41	119.7
C14—C13—H13	120.0	C40—C41—H41	119.7
C9—C14—C13	121.9 (8)	C41—C42—C43	119.6 (7)
C9—C14—H14	119.0	C41—C42—H42	120.2
C13—C14—H14	119.0	C43—C42—H42	120.2
C16—C15—C20	119.6 (7)	C42—C43—C44	120.6 (7)
C16—C15—Sn1	121.9 (5)	C42—C43—H43	119.7
C20—C15—Sn1	118.3 (5)	C44—C43—H43	119.7
C15—C16—C17	120.2 (8)	C43—C44—C39	120.6 (7)
C15—C16—H16	119.9	C43—C44—H44	119.7
C17—C16—H16	119.9	C39—C44—H44	119.7

### Hydrogen-bond geometry (Å, °)

**Table:**

*D*—H···*A*	*D*—H	H···*A*	*D*···*A*	*D*—H···*A*
N1—H1B···O2	0.86	2.02	2.683 (9)	133
N1'—H1'2···O4	0.86	1.99	2.63 (3)	130
